# Dynamic assessment of deltoid stiffness using shear wave elastography: a reliability study in healthy adults

**DOI:** 10.1016/j.xrrt.2025.08.018

**Published:** 2025-09-17

**Authors:** Jesse Seilern und Aspang, Frank L. Vazquez, Joanne Y. Zhou, Jaden Hardrick, Zaamin B. Hussain, Sarah M. Taub, Brittany R. Arnold, Michael B. Gottschalk, Eric R. Wagner

**Affiliations:** Department of Orthopaedic Surgery, Emory University School of Medicine, Atlanta, GA, USA

**Keywords:** Shear wave elastography, Deltoid stiffness, Reliability, Validity

## Abstract

**Background:**

Deltoid tension plays an important role in maintaining shoulder function. Understanding normative values is essential for accurately restoring deltoid mechanics in pathological conditions; however, there is a notable lack of data on this topic, particularly with respect to objective measurement methods. Shear wave elastography (SWE) is an ultrasound-based imaging modality that provides real-time quantitative assessment of muscle stiffness. This study evaluates the reliability and validity of SWE for measuring deltoid stiffness in different shoulder positions.

**Methods:**

A cross-sectional study was conducted on 21 healthy volunteers without shoulder pathology (8 males and 13 females; mean age 30.6 ± 5.6 years). Twelve SWE measurements of the middle deltoid were obtained for each side (left/right) and shoulder position (resting at side and 90° abduction) using a standardized measurement technique. Measurements were performed by 4 independent operators with varying levels of training and experience in ultrasound measurement (2 expert and 2 novice operators). Intraoperator and interoperator reliability were assessed using the median values for each measuring position, side, and measurer. Validity was assessed using Student's t-tests for resting vs. abducted positions, while reliability was evaluated with intraclass correlation coefficients (ICCs) and paired t-tests for side-to-side consistency.

**Results:**

The mean SWE values were 48.37 kilopascals (kPa) (left) and 47.64 kPa (right) at rest, and 158.48 kPa (left) and 155.45 kPa (right) in abduction. Tension was significantly higher in abduction compared to rest (156.97 kPa vs 48.01 kPa, *P* < .001), confirming construct validity by demonstrating SWE's ability to differentiate muscle stiffness across functional states. Interoperator reliability was good (ICC 0.79), and intraoperator reliability was also good (ICC >0.78). Reliability was also good between the 2 expert operators (ICC 0.878) and the 2 novice operators (0.797), as well as between expert and novice groups (ICC 0.762), indicating reliable measurements across experience levels. No significant difference was found between left and right measurements (*P* = .656).

**Conclusion:**

SWE is a reliable and valid method for quantifying deltoid muscle stiffness across functional states. Its reproducibility across operator experience levels and sensitivity to dynamic changes support its potential clinical utility in perioperative assessment and rehabilitation of shoulder conditions.

The deltoid is essential for shoulder motion and function, playing a central role in biomechanics, neuromuscular compensation, and recovery following injury or surgery.^3^^,^^7^ An optimized length–tension relationship is critical for maximizing muscle force generation, particularly in the deltoid. Surgical restoration of this native tension and vector is especially important in procedures like reverse total shoulder arthroplasty, where the surgeon modulates deltoid tension through implant lateralization and distalization.^8^ Proper tensioning is crucial, as both overtightening and under tightening can lead to complications such as acromial stress fractures and joint dislocations.^5^ Despite its clinical importance, there is currently no reliable, noninvasive method for measuring deltoid tension, and normative dynamic data in healthy individuals remain lacking. Establishing a reproducible means of quantifying deltoid stiffness could significantly inform surgical planning and rehabilitation strategies.

Shear wave elastography (SWE) is an emerging ultrasound-based imaging modality that enables quantitative assessment of skeletal muscle stiffness. By measuring the propagation velocity of induced shear waves, SWE provides objective, real-time evaluations of muscle mechanical properties.^11^ While this technology has demonstrated clinical utility in hepatology^6^ and oncology^13^, its application in musculoskeletal imaging is still evolving. Recent research has validated SWE for assessing rotator cuff and supraspinatus muscle stiffness, yet its reliability for measuring deltoid stiffness, particularly under dynamic and in vivo conditions, remains insufficiently explored.^2^^,^^12^

Examiner experience, probe and patient positioning, and pressure variations have been identified as potential sources of variability in SWE assessments, underscoring the need for standardized measurement protocols.^1^^,^^10^ Although SWE has shown moderate-to-high reliability for relaxed muscle assessments, existing studies have primarily focused on establishing age-dependent normative values for deltoid stiffness.^9^^,^^14^ However, its reproducibility in active contraction states remains unexamined, leaving a critical gap in functional musculoskeletal evaluations. Addressing this gap is essential for determining whether SWE can reliably quantify muscle stiffness across different functional states and examiner expertise levels, and in turn aids in establishing normative deltoid stiffness values that can inform perioperative planning, where precise modulation of deltoid tension affects outcomes.

This study evaluates the reliability and validity of SWE for measuring deltoid stiffness in different functional states. We hypothesize that SWE will demonstrate strong interoperator and intraoperator reliability and construct validity by distinguishing muscle stiffness between relaxed and abducted positions. We hypothesize that SWE will show good to excellent reliability across examiners and detect significantly greater stiffness during isometric contraction, validating its ability to measure dynamic changes. This study aims to establish SWE as a reliable tool for upper extremity assessment, supporting its clinical and research applications.

## Materials and methods

### Study design and participants

This study was an institutional review board–approved prospective reliability study designed to assess the interoperator and intraoperator reliability of SWE for evaluating deltoid muscle stiffness. A total of 21 healthy adult volunteers without shoulder pathology were recruited based on an *a priori* power analysis conducted using G∗Power 3.9.7 for an independent samples t-test, targeting a medium effect size (d = 0.5) and a desired power of 0.80.^4^ Each participant completed a questionnaire collecting demographic and characteristic variables, including age, sex, hand dominance, activity level, and history of prior shoulder injuries or surgeries (Table I). Activity was self-reported on a 5-tier activity scale: sedentary up to 10 lb occasional lifting with desk and household tasks and no sports; light up to 15 lb with light chores or recreation such as walking, light gardening, light swimming, or occasional road cycling; medium 20 to 30 lb with moderate exertion such as household carpentry, yoga, regular swimming or cycling, and gardening; heavy 50 lb or more with 1 to 2 weekly sessions of higher demand activities such as golf, tennis, rowing, basketball, off road cycling, light weight training, or heavier manual tasks; very heavy 100 lb or more occasional, or more than 50 lb frequent, or more than 20 lb constant lifting. Exclusion criteria included participants with a history of shoulder pathology (eg, rotator cuff tears, labral injuries), prior upper limb surgery, neurological conditions affecting muscle function, acute injuries within the past 6 months, or contraindications to ultrasound imaging. All participants provided written informed consent before data collection.

**Table I:** Patient demographics.

Variables	n (%)
Gender	
Male	8 (38)
Female	13 (62)
Age (mean ± standard deviation)	30.6 ± 5.6
Hand Dominance	
Right	17 (81)
Left	4 (19)
Sport/Activity Level	
Sedentary	0 (0)
Light	4 (19)
Medium	8 (38)
Heavy	5 (24)
Very Heavy	4 (19)

### Examiners and training

All measurements were performed by 4 examiners with varying levels of musculoskeletal ultrasound experience. Two of the examiners were advanced practice providers (APPs) with ≥4 years of ultrasound experience and daily use of ultrasound for diagnostics and therapeutics (APP1 and APP2), 1 was an orthopedic surgery resident (MD) with some musculoskeletal ultrasound exposure, but no daily use of ultrasound, and 1 was a medical student (MS) with minimal musculoskeletal ultrasound experience. APP1 and APP2 were categorized as experts while MD and MS were categorized as novices. Before data collection, all examiners underwent 1 h standardized training on SWE imaging protocols, including probe placement, data acquisition, and region of interest (ROI) selection. Training also emphasized alignment of the ultrasound probe with muscle fibers to minimize anisotropy effects and optimize image quality.

### Ultrasound equipment and shear wave elastography acquisition

All SWE assessments were performed using a GE LOGIQ ultrasound system (GE Healthcare, Chicago, IL, USA) equipped with a 9 MHz linear transducer. The same machine and transducer were used across all sessions to ensure consistency in image quality and acquisition parameters. System settings, including depth, gain, focal zone, and SWE color mapping, were manually adjusted and standardized prior to data collection. Specific probe positioning, patient setup, and acquisition protocol are detailed in the following Measurement Technique section.

### Measurement technique

**Figure 1:**
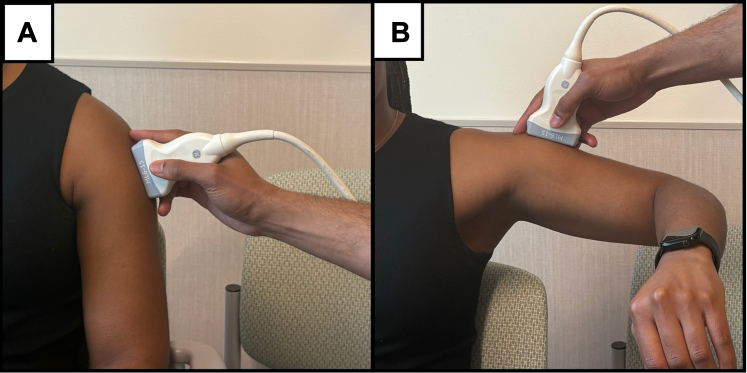
Standardized participant positioning and corresponding SWE images for deltoid assessment. (A) Resting position: shoulder in neutral alignment with the elbow supported to ensure deltoid relaxation. (B) Isometric contraction: shoulder abducted to 90°, held actively against gravity. (C, D) Corresponding SWE images acquired during rest and contraction, respectively, with ROIs selected from zones of optimal image quality as indicated by machine-coded white/yellow areas. *SWE*, shear wave elastography; *ROI*, region of interest.

All SWE measurements were acquired using a standardized protocol to ensure reproducibility across participants and operators. Participants were seated upright in a back-supported chair with both feet flat on the floor and shoulders exposed. Two shoulder positions were evaluated. In the resting condition, the arm was positioned neutrally at the side with the elbow supported on an armrest or pillow to promote deltoid relaxation. For isometric contraction, the shoulder was abducted to 90° and held against gravity while avoiding compensatory trunk motion (Fig. 1).

**Figure 2:**
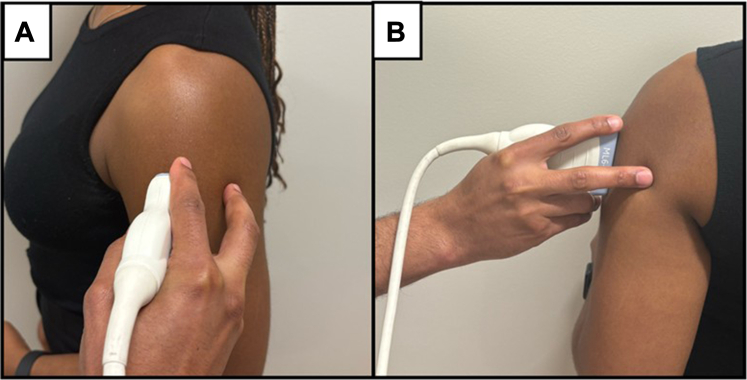
Standardized probe-holding technique for SWE acquisition. (A) side-view; (B) back-view. The transducer is held between the thumb and index finger, with the index and middle fingers anchoring it against the arm to maintain stability and minimize compression artifacts. *SWE*, shear wave elastography.

The probe was placed at the midpoint between the lateral acromion and deltoid tuberosity, aligned longitudinally with the middle deltoid fibers to minimize anisotropy. It was held between the thumb and index finger, with the index and middle fingers anchoring the transducer against the arm to ensure consistent contact and stability (Fig. 2). Generous ultrasound gel was applied to maintain acoustic coupling and minimize transducer pressure to avoid compression artifacts.

**Figure 3:**
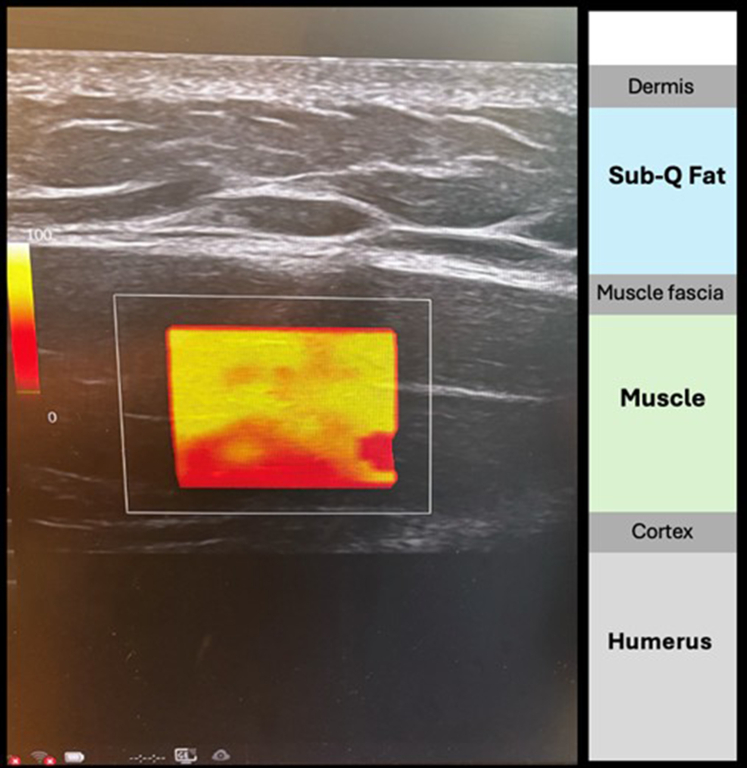
Representative SWE image of the lateral deltoid acquired with a 9 MHz linear transducer (GE LOGIQ). Anatomical layers are labeled, including subcutaneous tissue, deltoid muscle, and humeral cortex. Imaging was standardized at 3 cm depth with mid-muscle focal zone and optimized gain. The vertical color bar (*Left*) indicates machine-coded image quality, ranging from white (optimal) to red (poor). *SWE*, shear wave elastography.

Ultrasound parameters were standardized for all acquisitions: depth was set to 3.0 cm, the focal zone placed at mid-deltoid depth, and gain adjusted to optimize image clarity without oversaturation. The ROI was centered in the deltoid muscle belly, avoiding fascia, subcutaneous fat, and bone, guided by machine-generated color coding (Fig. 3).

Each examiner independently performed 12 SWE acquisitions per side and per position (rest and contraction). Representative images and ROI placement examples are shown in Figure 1. ROIs were selected from areas of highest image quality, identified by a color-coded quality map ranging from white (optimal) to red (suboptimal). Twelve nonoverlapping measurements were obtained from these optimal zones, and the median stiffness value, expressed in kilopascals (kPa), was recorded for analysis. Additional procedural tips for ensuring consistency and reliability are summarized in Table II.

**Table II:** Key tips to enhance consistency and accuracy of deltoid shear wave elastography acquisition, complementary to the standardized protocol.

Step	Tip
Patient position	Sitting upright, elbow supported (rest) or arm abducted to 90° (contraction) (Fig. 1)
Probe alignment	Long axis, parallel to muscle fibers
Stabilization grip	Thumb–index hold; anchor with index/middle fingers (Fig. 2)
Pressure control	Minimal force; use excess gel to avoid compression
ROI placement	Centered in muscle belly; avoid fascia and bone (Fig. 3)
Image settings	Depth 3.0 cm; focal zone mid-depth; gain optimized
Trial consistency	Use same location, angle, and arm support per trial

*ROI*, region of interest.

### Reliability assessment

To evaluate intraoperator reliability, each examiner performed repeated measurements on both shoulders of each participant. The contralateral side was used for within-subject comparison, rather than as a true control, to assess consistency across sides and reinforce measurement reproducibility. Interoperator reliability was evaluated by comparing measurements across all 4 examiners. If measurements from 2 examiners were compared against those of the other 2, the average of each pair's measurements was used for analysis.

### Statistical analysis

Statistical analyses were conducted using SPSS (version 29.0.0.0; IBM Corp., Armonk, NY, USA). Normality of data distribution was assessed using the Shapiro–Wilk test. Intraclass correlation coefficient (ICC) values were interpreted based on established guidelines, where values < 0.50 were considered poor reliability, 0.50-0.75 moderate reliability, 0.75–0.90 good reliability, and >0.90 excellent reliability.

A paired samples t-test was conducted to assess differences in SWE stiffness between rest and isometric contraction conditions. Additionally, linear regression analyses or student's t-test were performed to examine associations between shear wave velocity values and participant demographic characteristics, including age, sex, and hand dominance. One-way analysis of variance was used to compare shear wave velocity values between differing activity/sports level. A mixed-effects model was used to account for examiner variability in SWE measurements. Paired t-tests (or Wilcoxon signed-rank tests for nonparametric data) were used to compare stiffness values between rest and isometric contraction conditions, as well as between left and right measurements. Statistical significance was set at α = 0.05 for all analyses. Pearson Correlation Coefficient was used to evaluate the relationship between age and SWE values.

## Results

### Participant characteristics

A total of 21 healthy adult volunteers (8 males, 13 females; mean age = 30.6 ± 5.6 years) were included in this study. Participant demographics and clinical characteristics, including hand dominance, activity level, and prior history of shoulder injuries or surgeries, are summarized in Table I. No participants reported acute musculoskeletal conditions or contraindications to ultrasound imaging. The majority of participants were right-hand dominant (81%) and had medium to very heavy levels of physical activity (81%). There were no significant differences in elastography measurements when comparing demographic data (Table III).

**Table III:** Demographic data between relaxed and abducted measurements.

Variables	Deltoid relaxed (kPa)		Deltoid abducted (kPa)	
	Mean ± SD	*P* value	Mean ± SD	*P* value
Gender		.647		.628
Male	49.27 ± 8.79		160.98 ± 23.50	
Female	47.23 ± 10.30		154.50 ± 32.20	
Age (Pearson Correlation)	0.002	.993	−0.057	.806
Activity Level		.787		.396
Sedentary	46.65 ± 9.35		142.88 ± 34.21	
Light	49.05 ± 9.80		163.49 ± 30.34	
Medium	51.96 ± 4.97		147.08 ± 25.22	
Heavy	42.34 ± 13.88		170.36 ± 23.61	
Very Heavy	48.01 ± 9.58		156.97 ± 28.74	

*SD*, standard deviation.

### Interoperator reliability

Interoperator reliability for SWE measurements demonstrated good to excellent agreement, with ICC values ranging from 0.655 to 0.878 across all 4 examiners (Table IV). The highest agreement was observed in the resting position, while slightly lower ICC values were noted in the isometric contraction condition.

**Table IV:** Reliability measurements.

Reliability comparisons	ICC
MS	0.804
MD	0.830
APP1	0.861
APP2	0.776
MS vs APP1	0.820
MS vs. APP2	0.820
MD vs APP1	0.754
MD vs APP2	0.655
Novices: MS vs MD	0.797
Experts: APP1 vs APP2	0.878
Expert vs. novice group: APP1 + APP2 vs MS + MD	0.762
Overall interoperator reliability	0.787

*ICC*, intraclass correlation coefficient; *APP*, advanced practice provider; *MD*, surgical resident; *MS*, medical student.

### Intraoperator reliability

Intraoperator reliability analysis showed consistent measurements across repeated trials, with ICC values exceeding 0.78 for both arms. Intraoperator reliability for all operators is reported in Table IV. No significant differences were observed between left and right shoulder measurements (*P* = .656) (Table V), and no differences were found when comparing dominant versus nondominant sides (Table VI), supporting intraoperator consistency and the side-to-side reliability of the SWE technique.

**Table V:** Left vs. right deltoid measurements.

Condition	Left deltoid (kPa)	Right deltoid (kPa)	*P* Value
Overall	103.43 ± 60.35	101.55 ± 60.85	.656
Relaxed	48.37 ± 12.62	47.64 ± 11.42	.820
Abducted	158.48 ± 30.71	155.45 ± 8.04	.704

Data reported as mean ± standard deviation.

**Table VI:** Dominant vs. nondominant side measurements.

	Dominant side	Nondominant side	*P* Value
Deltoid relaxed	49.84 ± 14.74	47.77 ± 12.63	.628
Deltoid abducted	153.40 ± 36.46	155.58 ± 31.66	.837

### Differences in muscle stiffness between rest and isometric contraction

Mean SWE stiffness was significantly higher during isometric contraction compared to the resting condition (156.97 ± 28.74 kPa vs 48.01 ± 9.58 kPa, *P* < .001; Table VII). These findings indicate a physiological increase in muscle stiffness with activation, demonstrating SWE's sensitivity to dynamic musculoskeletal changes. The observed increase in stiffness during contraction was consistent across all examiners, with all measurers showing a significant difference between isometric contraction and rested measurements.

**Table VII:** Overall relaxed vs. abducted measurements.

	Deltoid relaxed	Deltoid abducted	*P* Value
Elastography measurements (kPa)	48.01 ± 9.58	156.97 ± 28.74	**<.001**

Data reported as mean ± standard deviation.

*P* value in bold indicates statistical significance.

### Influence of examiner experience on measurement consistency

A mixed-effects model analysis revealed no significant differences in SWE measurements based on examiner experience (*P* = .54), suggesting that standardized training protocols effectively minimized operator-dependent variability.

### Associations between shear wave elastography values and participant characteristics

Regression analysis showed no significant association between age or sex and SWE stiffness values (*P* > .05 for all models). Dominant arm measurements showed no significant differences when compared to nondominant measurements both when deltoid was relaxed, and when it was under tension (*P* = .628 and .837 respectively) (Table VI). Physical activity level was weakly correlated with SWE stiffness values in both resting and abducted positions (Unstandardized *B* = 3.26, *P* = .380), though this association was not statistically significant.

## Discussion

Deltoid tension is a critical determinant of shoulder function and surgical outcomes, particularly in procedures such as reverse shoulder arthroplasty, where optimal muscle tensioning is essential to avoid complications like periprosthetic joint dislocations or acromial fractures.^3^^,^^5^^,^^7^^,^^8^ However, despite its clinical significance, there is currently no reliable, noninvasive method to quantify deltoid tension, and normative values under dynamic conditions remain undefined. This study aimed to address that gap by evaluating SWE as a potential tool for objectively assessing deltoid stiffness in vivo.

Our findings demonstrate that SWE provides a reliable and reproducible method for quantifying deltoid muscle stiffness across functional states. Stiffness values were significantly higher during isometric contraction compared to rest, confirming SWE's sensitivity to physiologic muscle activation. Both interoperator and intraoperator reliability metrics were good to excellent, with minimal variability observed across examiners of differing experience levels. Additionally, no significant differences were identified between dominant and nondominant limbs or among demographic subgroups, supporting the method's consistency across healthy individuals. The absence of a correlation between age and deltoid SWE values in our cohort likely reflects the relatively narrow age range of healthy young adults included in this study. While height, weight, and occupational demands were not analyzed due to sample size limitations, future studies with broader demographic representation should explore their potential influence on muscle stiffness and SWE variability. However, collectively, these results establish normative dynamic stiffness benchmarks and highlight the feasibility of SWE as a noninvasive, real-time modality for assessing deltoid tension.

To assess the robustness of SWE across population variability, we evaluated the influence of demographic characteristics on stiffness measurements. Deltoid SWE values remained consistent irrespective of participant age, sex, or hand dominance. While a nonsignificant trend toward increased stiffness in individuals with higher physical activity levels was noted, the magnitude of variation was limited. These observations are consistent with previous studies demonstrating stable SWE metrics across healthy adult cohorts, with only modest declines in elasticity reported with aging.^9^ The stability of these measurements supports the development of broadly applicable normative reference values for future clinical use. The mean SWE values for the relaxed deltoid in this study were higher than those reported in prior literature. For example, Wang et al. reported average resting middle deltoid stiffness values of approximately 28-35 kPa using SWE in a healthy adult population.^9^^,^^16^ One possible explanation for this discrepancy may lie in differences in acquisition protocols and imaging software. In the present study, ROIs were selected exclusively from regions with optimal shear wave propagation, guided by the machine's real-time color-coded quality map. Measurements were accepted only if they achieved near 100% image quality, which may have led to consistently higher and more stable stiffness readings. This observation highlights the need for standardized ROI selection methods and cross-platform calibration when comparing SWE data across studies and systems.

A key outcome of this investigation was the high reliability of SWE when performed by both expert and novice operators. Interoperator and intraoperator agreement ranged from good to excellent (ICC 0.655-0.878), with no significant differences observed across experience levels. These findings corroborate prior reports of SWE's reliability in musculoskeletal applications when standardized acquisition techniques are followed.^1^^,^^10^ The consistency of repeated contralateral measurements further affirms the reproducibility of SWE and supports its integration into routine clinical workflows, irrespective of operator background.^15^

Importantly, SWE demonstrated a robust ability to distinguish between relaxed and active muscle states. Stiffness values increased significantly during isometric contraction across all raters, reinforcing construct validity and confirming SWE's responsiveness to functional muscular loading. This builds upon earlier studies that examined SWE under passive or static conditions,^12^^,^^16^ by extending the evidence base to dynamic assessment protocols. The method's reliability across a range of operator experience levels and its sensitivity to biomechanical changes position SWE as a promising tool for future investigation in perioperative evaluation, rehabilitation monitoring, and muscle function assessment in shoulder pathology.

This study has several limitations that warrant consideration. First, the sample was limited to healthy, young adult volunteers, which may restrict the generalizability of findings to older individuals or patients with shoulder pathology, including those undergoing reverse shoulder arthroplasty. Second, measurements were confined to 2 discrete shoulder positions without evaluating other functional ranges or dynamic movements that may be more reflective of clinical or athletic tasks. Third, while examiner training was standardized, complete blinding to the study hypotheses was not feasible, potentially introducing bias in measurement or interpretation. Fourth, SWE acquisition was limited to a single ultrasound system and probe configuration, which may not reflect performance across different platforms or equipment manufacturers. Finally, the scope of this study did not include assessment of longitudinal responsiveness of SWE to clinical changes, such as recovery after surgical intervention or rehabilitation, which is essential for establishing its utility in treatment monitoring.

## Conclusion

This study establishes SWE as a reliable, valid, and operator-agnostic modality for the quantification of deltoid muscle stiffness under both relaxed and active conditions. By demonstrating excellent reproducibility across examiners with varying levels of experience and confirming the sensitivity of SWE to physiological muscle activation, we provide foundational normative data for dynamic deltoid assessment. These findings support the integration of SWE into musculoskeletal evaluation protocols and highlight its potential utility in perioperative planning, rehabilitation monitoring, and biomechanical research involving shoulder function. Future work is warranted to validate SWE in surgical populations and to assess its impact on outcomes in procedures where deltoid tension plays a central role, such as reverse shoulder arthroplasty.

## Disclaimers:

Funding: No funding was disclosed by the authors.

Conflicts of interest: Eric R. Wagner receives consulting fees from Stryker, Smith and Nephew, Depuy-Synthes, and Acumed, and institutional research support from Konica Minolta. He also receives hospitality fees from Arthrex, Wright, Stryker, Integra, and Acumed. None of these are relevant to this manuscript. Michael B. Gottschalk, MD receives institutional support from Skeletal Dynamics, Acumed, and Arthrex; research support from Stryker and Konica Minolta; and serves in editorial and organizational roles including as a board or committee member of the American Society for Surgery of the Hand, associate editor for the Journal of Hand Surgery, and Surgical Techniques in Orthopaedics. He receives no royalties and none of these relationships are relevant to this manuscript. The other authors, their immediate families, and any research foundation with which they are affiliated have not received any financial payments or other benefits from any commercial entity related to the subject of this article.
